# Research to Translation: The Healthy Schools Toolkit and New Approaches to the Whole School, Whole Community, Whole Child Model

**DOI:** 10.1111/josh.12958

**Published:** 2020-11-12

**Authors:** Jason Q. Purnell, Nikole Lobb Dougherty, Emily K. Kryzer, Smriti Bajracharya, Veronica L. Chaitan, Todd Combs, Ellis Ballard, Allie Simpson, Charlene Caburnay, Timothy J. Poor, Charles J. Pearson, Crystal Reiter, Kelvin R. Adams, Michael Brown

**Affiliations:** ^1^ Associate Professor, Brown School|Director, (jpurnell@wustl.edu), Health Equity Works, Brown School, Washington University in St. Louis, One Brookings Drive, Campus Box 1196, St. Louis, MO 63130.; ^2^ Associate Director, (nlobbdougherty@wustl.edu), Evaluation Center, Brown School, Washington University in St. Louis, One Brookings Drive, Campus Box 1196, St. Louis, MO 63130.; ^3^ Project Coordinator, (emilykryzer@wustl.edu), Health Equity Works, Brown School, Washington University in St. Louis, One Brookings Drive, Campus Box 1196, St. Louis, MO 63130.; ^4^ Project Coordinator, (sbajracharya@wustl.edu), Center for Public Health Systems Science, Brown School, Washington University in St. Louis, One Brookings Drive, Campus Box 1196, St. Louis, MO 63130.; ^5^ Data Analyst, (vlchaitan@wustl.edu), Center for Public Health Systems Science, Brown School, Washington University in St. Louis, One Brookings Drive, Campus Box 1196, St. Louis, MO 63130.; ^6^ Research Assistant Professor|Assistant Director of Research, (tcombs@wustl.edu), Center for Public Health Systems Science, Brown School, Washington University in St. Louis, One Brookings Drive, Campus Box 1196, St. Louis, MO 63130.; ^7^ Assistant Professor of Practice|Director, (eballard@wustl.edu), Social System Design Lab, Brown School, Washington University in St. Louis, One Brookings Drive, Campus Box 1196, St. Louis, MO 63130.; ^8^ Program Coordinator for K‐12 Education, (a.simpson@wustl.edu), Social System Design Lab, Brown School, Washington University in St. Louis, One Brookings Drive, Campus Box 1196, St. Louis, MO 63130.; ^9^ Research Assistant Professor|Co‐Director, (ccaburnay@wustl.edu), Health Communication Research Laboratory, Brown School, Washington University in St. Louis, One Brookings Drive, Campus Box 1196, St. Louis, MO 63130.; ^10^ Publications Editor, (tpoor@wustl.edu), Health Communication Research Laboratory, Brown School, Washington University in St. Louis, One Brookings Drive, Campus Box 1196, St. Louis, MO 63130.; ^11^ (Retired) Superintendent of Schools, (cisroe@sbcglobal.net), Normandy Schools Collaborative, 8283 Glen Echo Drive, St. Louis, MO 63121.; ^12^ Director of Curriculum and Instruction, (creiter@normandysc.org), Normandy Schools Collaborative, 3855 Lucas and Hunt Road, St. Louis, MO 63121.; ^13^ Superintendent of Schools, (supt@slps.org), St. Louis Public Schools, 801 N. 11th Street, St. Louis, MO 63101.; ^14^ Deputy Superintendent, (michael.brown@slps.org), Office of Student Support Services, St. Louis Public Schools, 801 N. 11th Street, St. Louis, MO 63101.

**Keywords:** Whole School, Whole Community, Whole Child (WSCC) model, whole child, health communication, Healthy Schools Toolkit

## Abstract

**BACKGROUND:**

The Whole School, Whole Community, Whole Child (WSCC) model is an evidence‐based comprehensive framework to address health in schools. WSCC model use improves health and educational outcomes, but implementation remains a challenge.

**METHODS:**

Working with 6 schools in 2 districts in the Midwest, we used a mixed‐methods approach to determine the people, systems, and messages needed to activate WSCC implementation. We report on social network analysis and message testing findings and research translation to develop the Healthy Schools Toolkit.

**RESULTS:**

Social networks for both districts included more than 150 individuals. Both demonstrated network densities less than half of the desirable threshold, with evidence of clustering by role and minimal cross‐school relationships, posing challenges for WSCC implementation. Across stakeholder groups, messages that emphasize empathy, teamwork, and action were well‐received, especially when shared by trusted individuals through communication channels that align with stakeholder needs.

**CONCLUSIONS:**

The Healthy Schools Toolkit provides an example of a translational product that helps to bridge research with practice. With features that highlight 6 design principles, the toolkit provides complementary activities that schools and districts can use as they plan for integration of the WSCC model.

The 2014 development of the Whole School, Whole Community, Whole Child (WSCC) model^1^ was a historic collaboration between education and public health to advance student health and learning.^2^ WSCC merges components of the coordinated school health (CSH)^3^ and whole child approaches and identifies what is needed to achieve whole child health across 10 domains (Figure 1). Interventions conducted within such frameworks improve health and academic outcomes,^2^, ^4^, ^5^, ^6^, ^7^ which may help reduce inequality.^8^

Following the introduction of CSH and WSCC, several groups have proposed “key ingredients” that facilitate implementation.^9^, ^10^, ^11^, ^12^, ^13^, ^14^, ^15^, ^16^, ^17^, ^18^, ^19^, ^20^, ^21^, ^22^ Approaches that support implementation account for the integrated, collaborative nature of the frameworks and are comprehensive, building the infrastructure needed for sustainability.^23^ It is important to engage school health leaders and champions, generate buy‐in among stakeholders, and use a systematic approach when implementing school health initiatives. Despite these useful guidelines, sustaining school‐based health interventions remains challenging,^24^ and achieving full integration of WSCC is rare.^25^ This study addresses gaps in our understanding of WSCC implementation. We asked: If school health leaders and champions drive success, how do we identify and leverage those who are most influential? If buy‐in is key, what messages and communication strategies convey the importance of the work and activate stakeholders? And, if we use a systematic approach to implement WSCC, how do we ensure that this approach embeds the voices of all stakeholders and accounts for the complex nature of schools?

## Identifying and Leveraging Leaders and Champions

The importance of having champions who lead school health initiatives is well established; so, too, is building the organizational infrastructure that brings champions together for collaboration. For example, school health coordinators and health councils are critical to ensuring that health‐related work is prioritized.^18^, ^20^, ^21^, ^22^, ^26^, ^27^, ^28^ According to Rasberry et al., school principal commitment signals to the school community that health priorities are integrated into policies and practices.^21^ Others report the importance of building a team of champions across a school—including those “in the trenches”—among whom leadership and responsibility can be distributed.^26^, ^27^

Identifying school health leaders who have influence and understanding how those leaders share information with others has received little attention. Social network analysis (SNA), a set of methods from the social sciences, can provide insights.^29^, ^30^ SNA is particularly useful when considering relationships between and among individuals in a social network. The application of SNA in education and public health is not new. Studies in both fields underscore the value of strong social networks in educational reform and public health program implementation.^30^, ^31^, ^32^, ^33^, ^34^, ^35^, ^36^, ^37^, ^38^, ^39^, ^40^ SNA has not been used to address the challenge of implementing a comprehensive approach to school health like WSCC.

**Figure 1:**
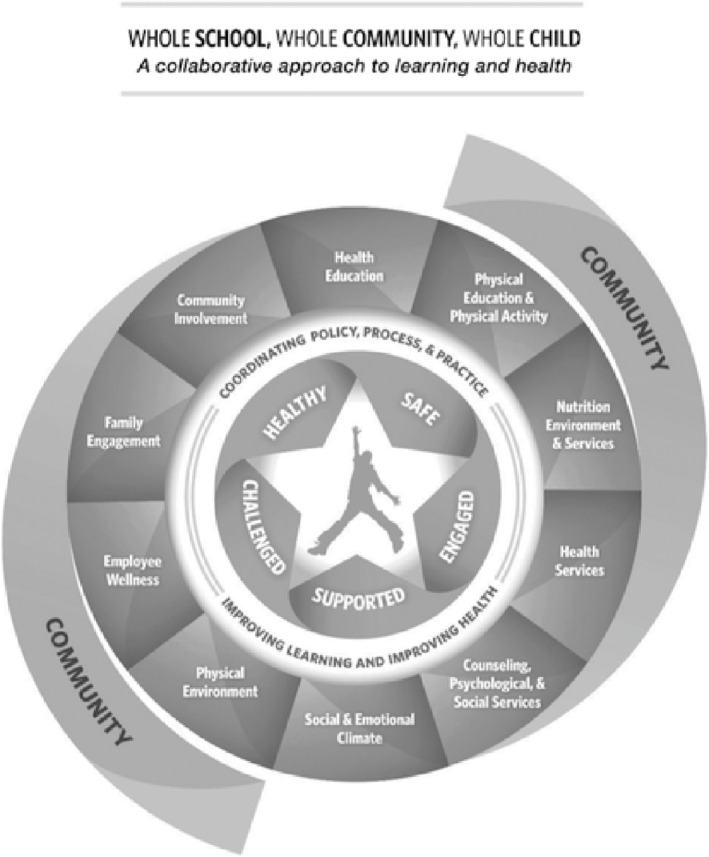
The Whole School, Whole Community, Whole Child Model

## Communicating Effectively

WSCC implementation also requires the buy‐in of stakeholders, especially school and district leaders.^40^ Multiple strategies for securing this support have been described, including: (1) sharing research about the relationship between learning and health; (2) articulating how creating a healthy school aligns with school and district priorities; (3) inviting administrators to join a school health council or meeting; and (4) spreading school health messages publicly via various channels.^41^ Health communication science, or the study and use of communication strategies to inform and influence decisions and actions to improve health,^42^ is essential to any public health campaign. Application of health communication research to determine the most effective messaging strategies will benefit efforts to establish buy‐in across stakeholder groups and achieve broader dissemination and implementation of the WSCC model.

## Changing and Aligning Systems

The WSCC model (Figure 1) acknowledges the importance of aligning systems to support healthy school environments. Research suggests that a systematic approach to planning and implementation is beneficial.^15^, ^19^, ^20^, ^21^, ^22^, ^41^ A synthesis by Hunt et al.^19^ recommended a 10‐step process whereby teams identify and prioritize needs, align health outcomes with academic priorities, and develop, implement, and monitor action plans. This process is similar to one described in the National Association of Chronic Disease Directors' (NACDD) publication, *The Whole School, Whole Community, Whole Child Model: A Guide to Implementation*.^41^ These and other examples emphasize the importance of collaboration with communities and families.^20^, ^21^, ^27^

The benefit of these approaches is that they are easily understood and actionable. Conceptualizing WSCC promotion as a complex systems problem and applying systems modeling tools to solving implementation challenges would provide additional benefits.^43^, ^44^, ^45^, ^46^, ^47^ As a participatory methodology, a systems approach also allows for the diverse perspectives of education stakeholders to be explored. Such application of systems approaches is considered by Ballard et al.^48^ in this special issue.

## Current Study

The current study assumes that frameworks like WSCC do not implement themselves; rather, people with influence, who communicate effectively, and who are embedded within functional systems, implement frameworks. Our purpose was 2‐fold: First, we deployed research methods within school systems for identifying and leveraging people, promoting messages, and changing systems in service of healthy schools. Second, we translated our experience into the Healthy Schools Toolkit, a step‐by‐step guide designed to help educators replicate the methods that we used in their local context. This work was completed as part of Together for Healthy and Successful Schools (THSS), an initiative of the Robert Wood Johnson Foundation (RWJF) that aimed to advance real‐world approaches that support health in schools. This paper describes the objectives of the research study and the translation processes used to develop the toolkit, presents key findings, and reflects on insights for the school health field.

## METHODS

### Participants and Recruitment

We established research partnerships with 2 school districts serving high‐need student populations in the Midwest. Districts were chosen because they: (1) provided test cases with potentially generalizable challenges, opportunities, and needs; (2) allowed for a focus on equity, one of the central goals of the THSS initiative; and (3) had previously established partnerships with the research team. District A is smaller, with roughly 3500 students; District B has over 20,000 students.

To determine necessary conditions for WSCC implementation across the K‐12 spectrum, the research team chose one elementary, middle, and secondary school in each district. School selection considered several factors: (1) near‐average enrollment for its district; (2) locations in relatively less socioeconomically advantaged neighborhoods; (3) community partner experiences; and (4) school leadership. Each district identified district‐ and building‐level liaisons who worked closely with the research team to coordinate research activities and facilitate connections with stakeholders.

The project included multiple research activities with overlapping participant samples. The target sample for activities included district leaders, building administrators, staff members, teachers, parents, and community partners who were identified as key stakeholders and opinion leaders. Occasionally, the target sample was expanded to include any member of the district or school community and high school‐aged students.

The study addressed 4 objectives through 5 methods: (1) a *stakeholder survey* to assess perceptions of the importance of health in schools, the impact of WSCC components on student success, and the barriers to and facilitators of promoting WSCC; (2) *key informant interviews* to further assess perceptions, barriers, facilitators, and priorities; (3) a *social network analysis* (SNA) *survey* to analyze the characteristics of school‐ and district‐level networks and identify core influencers who could support WSCC implementation; (4) *message testing* to uncover key message themes, strategies, and messengers to promote WSCC; and (5) *group model building* to graphically represent the factors that interact to produce healthy schools. In this paper, we detail the methods and results related to SNA and message testing. Ballard et al.^48^ report on group model building.

#### *Social network analysis*

An iterative roster development process was used to identify key stakeholders. Liaisons completed a roster development survey, identifying district leaders, teachers, administrators, community partners, parents, and students who were highly visible, active, or well‐informed. Each recommended individual was then asked to recommend others. This process continued until a representative sample was produced and the roster approached saturation. School rosters ranged between 40 and 75 participants. The variety in roster size was generally proportional to staff size (approximately 35–85 staff members) and student enrollment (approximately 180–615 students).

#### *Message development and testing*

Message testing interviews were targeted across various roles. For each school, interviews were sought with one building administrator, teacher, staff member, and parent; with each district, interviews with one district leader, one board member, and 2 community partners were sought. Interviewees were identified through 2 channels: (1) a subset of those highly recommended through roster development; and (2) parents and guardians identified by liaisons as being representative of each school's population.

Recruitment for all research activities took place between October 2017 and June 2018. Participants were invited through phone calls, emails, and school and community events. School and district leaders and liaisons facilitated many connections and helped to coordinate in‐person recruitment and data collection.

### Procedures

#### *Social network analysis*

Social network analysis (SNA) takes place first through roster development (described above) and then a network survey. Network survey participants were asked about 2 aspects of their relationship with individuals listed on the roster for their school and district: (1) their frequency of contact with each person listed, and (2) their level of trust in information shared by them. When it was not possible to complete the survey online, the research team arranged for participation by phone, by mail, or in person. A template (https://wustl.app.box.com/s/i7dvfp29ghdewh749h0v34ljxkhbmcjk) for preparing an SNA network survey is available via the Healthy Schools Toolkit website.

#### *Message development and testing*

Message testing began with the research team completing an environmental scan of messages based on WSCC, including those from the US Centers for Disease Control and Prevention (CDC), RWJF, and several school health advocacy organizations. Approximately 36 messages that targeted different audiences were developed.

Messages were tested during 2 rounds of interviews. Interviews were designed to identify messages that were interesting, clear, easy to understand, and memorable, and that stimulated self‐reflection and were personally relevant.^49^, ^50^ A 2‐person team conducted interviews at school or district buildings. Each interview lasted approximately 45 minutes. A list of messages tested in Round 1 (https://wustl.app.box.com/s/35zd93tbwmgfv0obfpl7w127i0mqo80l) and Round 2 (https://wustl.app.box.com/s/8o6j28cnrodej2715dli7udw7i0dsxdh) of message testing are available on the Healthy Schools Toolkit website.

The first round consisted of questions to understand what a “healthy school” meant to participants and how they perceived their role in supporting a healthy school. Following this, participants completed a card sort activity in which they reviewed and provided detailed feedback on a set of 15–20 messages. Each participant was presented with messages targeted for their audience and also general healthy school messages for other audiences. During the activity, participants were asked to identify the messages they liked and disliked most and why. Among their most‐liked messages, participants were asked who most needed to hear the message, who might best deliver it and through what communication channel, and which messages might inspire action. The protocol for the second round of interviews was similar to that of Round 1. Templates for preparing interview guides for Round 1 (https://wustl.app.box.com/s/6cepcocwgc0dczi30us4c0rem1mx169e) and Round 2 (https://wustl.app.box.com/s/q64c3q874esevwp9v09ek0hmdcymbsp6) of message testing are available via the Healthy Schools Toolkit website.

### Data Analysis

#### *Social network analysis*

Survey data were analyzed using the statistical software R.^51^ Trained social network analysts used 2 common SNA techniques, visualization and description, to understand network structures and to identify core influencers.^52^ To characterize networks, the following metrics were calculated: (1) *overall network density* (how connected each network was; if everyone communicated frequently and highly trusted one another as sources of school information, the density would equal one, or 100%; if no one communicated or trusted information, density would equal zero); (2) *average in‐degree* (the average number of times a person was identified as highly communicative and trusted); and (3) *average role diversity of ties* (to identify individuals who were well‐connected with others in different roles, the team looked at how diverse, in terms of 6 roles, each individual's network ties were, and averaged this for each network).

To identify *core influencers*, analysts selected individuals who: (1) were involved in frequent (at least monthly) communication and in trusting relationships (at least one of the individuals reported moderate or high trust between the 2); and (2) ranked among the top 4 individuals in their role as having the most reported influence (in smaller schools, fewer than 4 people were eligible for each role). R was used to create network visualizations, where nodes (individuals) are (1) represented by different shapes that reflect the individual's role, and (2) are sized by the number of relationships or ties (lines) each has.

#### *Message development and testing*

Following the first round of interviews, trained qualitative analysts reviewed interview notes to refine existing messages and develop both new messages and 2 brief stories that reflected the most popular message themes from Round 1 to test in Round 2. Following the second round, analysts applied a hybrid deductive/inductive approach to identify generally liked and disliked messages and key themes and to summarize message preferences by stakeholder group.^53^, ^54^, ^55^

### Data Synthesis and Translation

Data synthesis and translation occurred in 2 phases for 2 audiences: (1) the districts with whom research was conducted and (2) a national audience of school health and education leaders interested in building healthy schools. First was the development of district‐specific written reports and presentations slides that summarized findings and provided recommendations to guide WSCC implementation. District meetings were held with stakeholders to gather feedback, which was incorporated into districts' final reports.

The second phase led to the development of the Healthy Schools Toolkit^56^ and included: (1) synthesizing information across district reports; (2) convening regional and national advisory councils to guide toolkit development and dissemination plans; (3) applying advisory councils' feedback to the toolkit content and design; and (4) testing elements of the toolkit with education stakeholders attending a regional work session. Throughout these processes, a graphic design firm, consultant writers, and a dissemination manager experienced in product translation for non‐academic audiences were utilized.

The regional and national advisory councils were convened with support from the Center for Society and Health at Virginia Commonwealth University, which has extensive experience engaging diverse stakeholders in the education and health sectors. The regional advisory council included liaisons and district leaders from the 2 study districts, representatives of the research team, and 9 regional organizations. The national advisory council included 24 experts representing health and education advocacy organizations, research and academic institutions, and national groups representing specific populations. Both advisory councils met quarterly, beginning February 2018 and concluding September 2019.

During toolkit development (between January and June 2019), the project team facilitated a preview of one module of the toolkit with stakeholders at a regional work session on healthy schools. Following a presentation on WSCC and key findings from the study, small groups of attendees read the first few sections of one module, engaged in discussion, and completed “Action Items” based on a school health‐related scenario they were assigned (eg, “You are new to your school and notice that a lot of students come to your classroom hungry, even though the school provides free breakfast”). A total of 27 attendees representing 15 regional districts completed evaluations of the module's usability and applicability. Recommendations from this group and the advisory councils were used to finalize the toolkit and supplementary resources.

## RESULTS

### Social Network Analysis

The networks described contain those individuals tied to others in relationships that were highly communicative and between people who trusted each other as sources of school information. Table 1 compares the distribution of roles among members of each district's network and outlines demographics of SNA survey respondents. District A's and B's social networks include 150 and 168 individuals, respectively. The largest stakeholder group in District A's network is teachers (31%) followed by staff (27%) and parents (16%). Network membership in District B consists of mostly teachers (27%) followed by parents (21%) and community partners (20%).

**Table 1:** Role Distribution of Social Network Members and Demographic Characteristics of SNA Survey Respondents

	District A	District B
Characteristics	N (%)	
Role		
Administrator	11 (7)	7 (4)
Community partner	15 (9)	33 (20)
District leader	13 (8)	16 (10)
Parent	23 (16)	36 (21)
Staff	42 (27)	31 (18)
Teacher	46 (31)	45 (27)
Sex		
Female	80 (53)	71 (42)
Male	21 (14)	19 (11)
Not reported	49 (33)	78 (46)
Time at school		
Less than 1 year	16 (11)	11 (7)
From 1 to 3 years	26 (17)	22 (13)
From 3 to 5 years	14 (9)	22 (13)
5 years or more	47 (31)	39 (23)
Not reported	47 (31)	74 (44)
Income		
Below $35 k	10 (7)	22 (13)
$35 k‐$50 k	19 (13)	21 (13)
$50 k‐$75 k	23 (15)	22 (13)
$75 k‐$100 k	20 (13)	8 (5)
$100 k‐$150 k	15 (10)	7 (4)
$150 k and above	9 (6)	6 (3)
Not reported	54 (36)	82 (49)
Level of education		
High school diploma/GED	4 (3)	3 (2)
Some college	9 (6)	13 (8)
2‐year degree/Vocational training	6 (4)	6 (4)
4‐year degree	24 (16)	24 (14)
Graduate/Professional degree	58 (39)	44 (26)
Not reported	49 (33)	78 (46)
Race and ethnicity		
Non‐Hispanic Black or African American	70 (47)	52 (31)
Non‐Hispanic White or Caucasian	21 (14)	28 (17)
Other or multiple races/ethnicities	6 (3)	9 (13)
Not reported	53 (35)	79 (47)

District A and B's social networks include 150 and 168 individuals, respectively. Of these, the research team requested individuals to complete 169 and 172 SNA surveys (some individuals were asked to complete multiple surveys for different schools). In total, 116 surveys were completed for District A, and 98 surveys for District B, for response rates of 69% and 57%.

#### *Network characteristics*

Table 2 shows key characteristics of the districts' networks. District A's network demonstrates a higher level of connectivity, with members in contact with and trusting an average of 40 people, compared to just under 20 people for District B. Cross‐role interactions, or *average tie diversity*, and *network density* also differ between the networks (Table 2). On average, members of District A's network are connected with individuals who represent 5 (out of 6) roles (average tie diversity is 5.1), whereas members of District B's network are connected with others in about half of the possible roles (average tie diversity is 3.4). This indicates that, in District A, a typical stakeholder communicates frequently with individuals in the same role and others in 4 additional roles; in District B, typical communication is with individuals in the same role and others in 2 additional roles. District A's network density (0.13)—or the proportion of existing ties versus all possible ties—is double that of District B (0.06).

**Table 2:** Characteristics of Social Networks

Characteristics	District A	District B
Network size (the number of unique individuals within a network)	150	168
Response rate (the number of individuals who responded to the social network survey out of those who were invited)	69% (116/169)	57% (98/172)
Network ties (the total number of relationships that connect all individuals in the network)	4510	1596
Average network ties (the average number of relationships that connect an individual to others in the network)	40	19
Tie diversity (the average number of cross‐role relationships that connect an individual to others in the network)	5.1	3.4
Network density (the number of existing ties compared to all possible ties)	0.13	0.06

**Figure 2:**
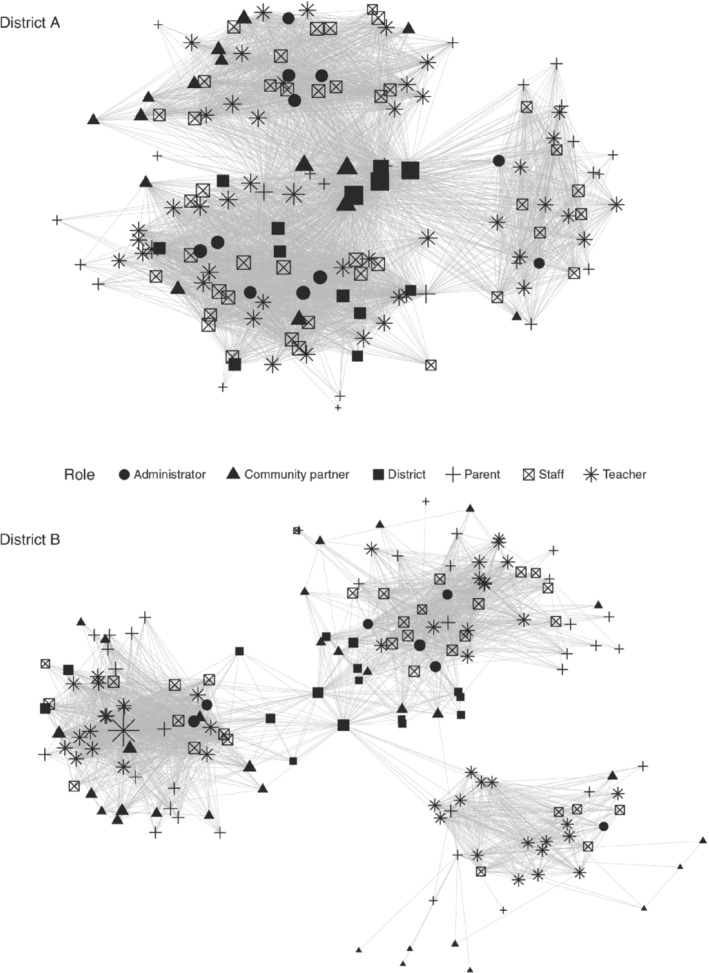
Social Network Maps

These patterns are evident in the social network maps presented in Figure 2, which show the combined structure of contact and trust in District A and B. Network maps are laid out where connected nodes are centered toward the middle. Nodes that are more isolated with fewer ties are toward the outer edges. The 3 clusters of nodes in each figure represent the school networks. Ties in Figure 2 represent each node's *in‐degree* (described above).

**Figure 3:**
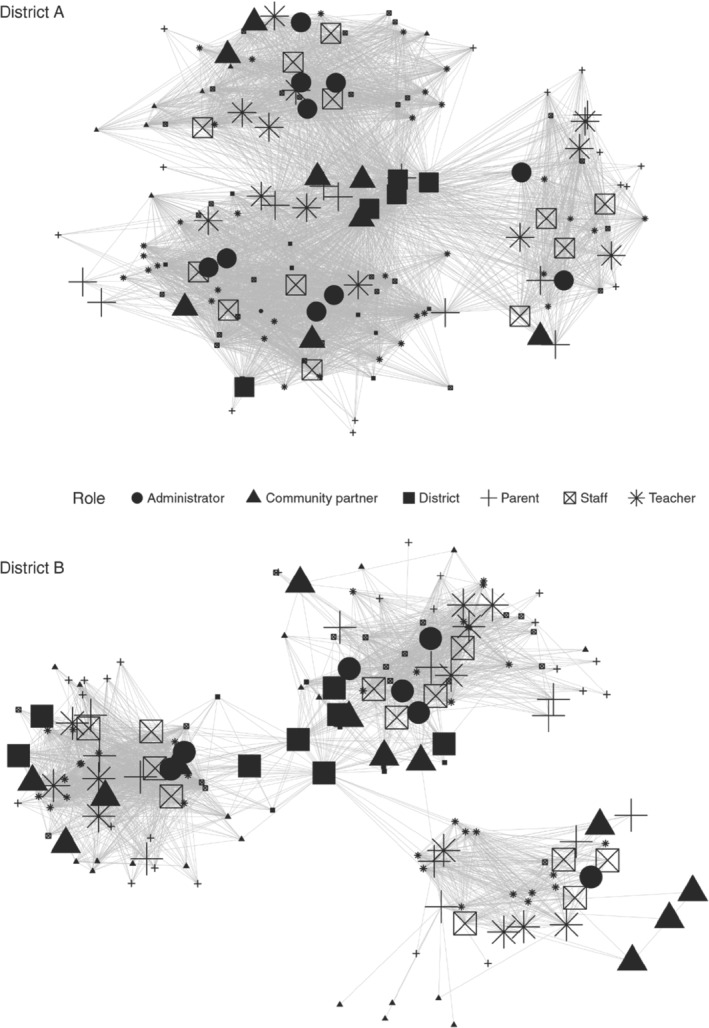
Social Network Maps with Core Influencers

An important area of focus for District A's network (Figure 2) is the middle, where the nodes that serve to connect the 3 school buildings lie. Most of these individuals are community partners, joined by some district leaders and parents. For District B's network, only 2 district leaders are identified as connecting the schools.

#### *Core influencers*

Core influencers (described above) are indicated in Figure 3. Here, the larger node reflects that the individual is a core influencer. A similar number of individuals were identified as potential core influencers in District A (N = 57) and District B (N = 63).

### Message Development and Testing

Across the 2 rounds, 29 individuals from District A and 27 individuals from District B participated in message testing interviews. Table 3 provides the demographic characteristics of interviewees. Participation across roles was relatively balanced in both districts. Below are message themes, messengers, and messaging strategies preferred by interviewees.

**Table 3:** Demographic Characteristics of Message Testing Interviewees

	District A	District B
Characteristics	N (%)	
Role		
Administrator	3 (10)	4 (15)
Community partner	5 (17)	3 (11)
District leader	4 (14)	3 (11)
Parent	4 (14)	7 (26)
Staff	6 (21)	4 (15)
Teacher	7 (24)	6 (22)
Sex		
Female	23 (79)	20 (74)
Male	6 (21)	7 (26)
Income		
Below $35 k	3 (10)	9 (33)
$35 k‐$50 k	4 (14)	5 (19)
$50 k‐$75 k	9 (31)	7 (26)
$75 k‐$100 k	6 (21)	2 (7)
$100 k‐$150 k	4 (14)	2 (7)
$150 k and above	3 (10)	2 (7)
Level of education		
High school diploma/GED	2 (7)	1 (4)
Some college	1 (3)	5 (19)
2‐year degree/Vocational training	0 (0)	3 (11)
4‐year degree	9 (31)	4 (15)
Graduate/Professional degree	17 (59)	13 (48)
Not reported	0 (0)	1 (4)
Race and ethnicity		
Non‐Hispanic Black or African American	22 (76)	17 (63)
Non‐Hispanic White or Caucasian	5 (17)	7 (26)
Other or multiple races/ethnicities	2 (7)	3 (11)

Total N_DistrictA_ = 29; Total N_DistrictB_ = 27.

#### *Messages that resonate with stakeholders*

Four categories of message themes were preferred, including messages that: (1) connect education with health; (2) express empathy for students and parents; (3) emphasize teamwork; and (4) suggest specific actions.

1. ***Connecting education with health***. In general, interviewees saw schools as playing an important role in supporting and promoting students' health and favored messages that broadly connect the role of health to education (Table 4). They noted students unable to focus and learn because their physical and mental health needs went unmet. As one staff member stated, “*It is one of the most basic things you can tackle. Students in our district are dealing with so much. It can affect their ability to focus*.” Interviewees also noted educators being unable to fully engage with students, teach effectively, or support students in other ways because of their own physical and mental health issues.
2. ***Expressing empathy***. Interviewees in both districts favored messages that expressed empathy for students and parents and acknowledged the role that social and economic challenges play in their lives. Reading empathetic messages made interviewees think about their own challenging experiences and encouraged an understanding of the struggles students and their families confront. As one teacher stated: “*That is what motivates [students]. If they don't think that you care, they don't think that you know anything about them, they think that it's just another day*.” Interviewees reported that messages that articulate care and a willingness to meet students where they are help create support for healthy schools.
  Different empathy‐focused messages resonated with different groups in District A and B (Table 4). In general, teachers and staff in both districts liked a broader number of messages, while school board members liked fewer messages.
3. ***Emphasizing teamwork***. Messages that emphasized teamwork were also appealing. Many referred to the common adage, “*It takes a village*,” when speaking about how they support students, such that messages that encourage teamwork reduce the pressure on any one individual or group. As one teacher stated: “*A lot of times, our parents feel like they're alone, or they've just given up. They need to know that we are all in this together*.” Interviewees from District A and B preferred different teamwork‐focused messages (Table 4).
4. ***Suggesting specific actions***. Messages that included suggestions for a specific action were also preferred. Across both districts, messages suggesting specific actions were seen to help “empower” teachers and encourage parent involvement. There was less consistent agreement across roles about preferences for action‐oriented messages (Table 4).

**Table 4:** Most‐Liked Messages by Target Audience

Messages that …	Parents	Teachers	Staff	Administrators*	Community Partners	Board Members
Connect Education with Health^30^						
A healthy school is one that nurtures and supports every aspect of a student's health—physical, social, emotional, and cognitive health. This means getting kids active and eating healthy foods. School buildings and grounds that make kids and staff feel motivated and ready to learn. Teachers who support students and serve as positive role models. A welcoming place for parents and families to learn about how they can help their kids succeed. And working with a community that promotes learning.	•	•	•	•	•	•
	◆	◆	◆	◆	◆	◆
Every student deserves a chance to succeed, no matter who they are, where they are from, or how much money their family makes. More than half of our nation's public school students live in poverty. Schools can help kids overcome challenges and give the skills they need to succeed in life. But schools need resources, and families and communities need to be involved so all students have supportive network at school, at home, and in between.	•	•	•	•	•	•
	◆	◆	◆	◆	◆	◆
In order to graduate ready to succeed in an increasingly competitive workforce/in life, young people need a well‐rounded set of skills—not just book smarts, but strong interpersonal relationships, social skills, and self‐confidence, as well as the ability to work effectively in teams. Students need to learn these skills at home and during the school day.	•	•	•	•	•	•
	◆	◆	◆	◆	◆	◆
Healthy, safe, and nurturing school environments ensure that all our children are ready to learn. When children are healthy—physically and emotionally—and are surrounded by caring teachers and adults who support them, they are able to focus on what they are learning.	•	•	•	•	•	•
	◆	◆	◆	◆	◆	◆
Academic achievement is important, but it's only one piece of the puzzle. In order to help all students succeed in life, we should support an approach in schools that nurtures a student's health and well‐being to help them grow into successful, well‐rounded adults.	•	•	•	•	•	•
	◆	◆	◆	◆	◆	◆
Express Empathy for Students and Parents						
Meet them where they are. Knowing their challenges can help you meet yours.	•	•	•			
	◆	◆	◆			
Children need to know you care.	•	•	•	•	•	
	◆	◆	◆	◆	◆	
Treat them like your own. Care about their health and wellness as you would if they were your own children.	•	•	•	•		
		◆	◆	◆	◆	
Helping kids with their emotional needs and healthy thinking are essential for creating a safe and productive place to learn.	•	•				
		◆	◆			
Parents need to know you care.	•	•	•	•	•	
	N/A					
Healthy in body and mind. Mental health services are essential for creating a healthy, safe place to learn.	N/A					
				◆	◆	◆
Emphasize Teamwork among Stakeholders						
Teachers: The First Responders. To care for the whole child, schools need experts to whom teachers can refer kids with problems beyond the classroom.	•	•				
				◆		◆
Creating a healthy school is a team effort.	•	•	•	•	•	•
	N/A					
Parents who feel welcome in school can help make a difference for students.	N/A					
		◆	◆	◆		
You're not alone. Administrators. Community. Parents. Teachers. We're all working together to help our children succeed.	N/A					
	◆	◆	◆	◆	◆	◆
Strong schools, strong community. Volunteer at your [kids'/local] school to help make a difference.	N/A					
	◆				◆	◆
School safety: It's more than a metal detector. Effective safety includes existing programs and resources as part of a comprehensive, whole‐school approach.	N/A					
				◆	◆	◆
Healthy schools, better together. Administrators. Community. Parents. Teachers. If we work together, we can help all our children succeed.	N/A					
	◆	◆	◆	◆	◆	◆
Suggest Specific Actions						
Get inside the backpack. Showing your child you care about what happens in school will make you both proud.	•					
	N/A					
Mobilize for healthy kids. Make healthy policies and the resources for them a priority.	•	•	•			
	◆				◆	
Mobilize for healthy kids. You know that health and education go hand in hand. Volunteer!	N/A					
		◆	◆			
Mobilize for healthy kids. Share your experience with others.	N/A					
				◆	◆	◆

* Administrators include School Administrators and District Leaders.

• = District A; ◆ = District B; N/A = not a “most liked” message.

#### *Messengers to engage stakeholders*

Receiving information from multiple people within the school and district was preferred. In both districts, interviewees shared that school administrators are the “go‐to” source of building‐level information for teachers, staff, and community partners. Instructional coaches are also important messengers for teachers.

Parents rely heavily on teachers and support staff for information about their children and services and on district leaders for information about the district and community. Community leaders and local organizations are also effective messengers if they are well‐established in the district.

#### *Messaging strategies to reach stakeholders*

Information is circulated inside schools and across the larger system about activities, initiatives, instruction, wellness, and community‐engagement campaigns. Interviewees reported they value the use of diverse communication strategies and recommended that districts leverage strategies to engage existing communication networks. Stories and testimonials were seen as particularly powerful strategies for sharing messages and are most effective when they are short, use plain language, and are presented using digital formats and platforms. Table 5 summarizes the advantages and disadvantages of frequently used strategies. As with other messaging‐related results, the effectiveness of communication media depends on audience, message content, and purpose.

**Table 5:** Message Testing Interviewees' Descriptions of Advantages and Barriers Related to Preferred Communication Methods

Preferred Communication Methods	Advantages	Barriers
E‐mail	Widely used within the school system Convenient to get information Could be used alone or in combination of other channels Easy to archive	Difficult to keep up with high volume Information overload/fatigue Passive form of communication Less opportunity for dialogue and relationship building
Text message	Widely used within the school system Efficient ways to reach most audiences Possible to set up automated text messages and phone calls for brief messages such as event reminders	Some parents do not have access to consistent mobile phones Difficult to engage with high volume of automated messages Passive form of communication Less opportunity for dialogue and relationship building
Face‐to‐face conversations	Used when necessary to coordinate school activities internally and share information from the school or district with community members and parents Creates opportunity for two‐way dialogue and active relationship building	Takes time to schedule and to meet May need additional meeting preparations (eg, create an agenda and meeting goals, etc.)

### Toolkit Translation

#### *Advisory councils*

Advisory council feedback was used to develop a list of design principles for the toolkit. These principles emphasized that the toolkit be: (1) *accessible*: use plain language and effective data visualization; (2) *complementary*: intentionally connect to other WSCC resources; (3) *actionable*: provide instructions and describe the “do‐ability” of the methods; (4) *modular*: provide information so anyone can accomplish something valuable in the time that they have; (5) *personalized*: create individualized guidance for how to use the methods given one's local setting and role; and (6) *responsive*: reflect the lived experience of educators, especially through the use of storytelling.

#### *Regional facilitated review*

A majority of respondents (N = 27) who evaluated a toolkit module as part of a facilitated preview indicated that they strongly agreed that information in the toolkit was easy to understand (85%); graphics were interesting (85%); activities were relevant to educators (81%); and information was useful (74%) and could help educators be effective (78%). A majority of stakeholders also strongly agreed that they would use the toolkit module (67%) and recommend it to their colleagues (56%).

#### *Toolkit features and supplementary products*

The finalized Healthy Schools Toolkit consists of 3 modules on People (social network analysis), Systems (group model building), and Messages (message testing). Each module features elements, which were developed to align with guiding *design principles*:

- A Quick Start Guide that describes the steps for each method (*accessible, modular*)
- An Options table that outlines a range of activities that can be completed and the human and knowledge resources needed to achieve each (*modular, personalized*)
- A Possible Impacts story that provides illustrative stories about how methods can impact school environments (*responsive*)
- Action Items that serve as opportunities for readers to practice recommended techniques (*actionable)*
- A case study that features in‐depth accounts of the lessons learned in the study districts (*responsive)*

A suite of supplementary resources were also developed, including 2 animated videos that feature district leaders' perspectives on how the methods highlighted in the toolkit impacted their efforts to build healthy schools (*responsive*), a compendium of expert‐recommended readings, websites, and other external resources (*complementary*), and over 30 customizable templates to facilitate key steps of the toolkit (*actionable*). These and the toolkit are free and available on a website (*accessible*) designed and hosted by Health Equity Works at Washington University in St. Louis.^56^

## DISCUSSION

The methods applied in this study and represented in the Healthy Schools Toolkit provide new approaches to understanding challenges to WSCC implementation. Through SNA, study districts gained a deeper understanding of how individuals in their school communities communicate and relate with one another. This emphasis on the *relational* aspects of how new information and practices are spread is an important feature of SNA. Organizations, including schools, often attempt to communicate new information without taking into account the relationships that allow for that information to reach everyone and be accepted by them. Finnigan and Daly^31^ note that schools “push” information through staff meetings, professional development sessions, or other trainings. When these activities do not result in successful implementation, the assumption may be that the information is flawed, but it is possible that messengers are not trusted or that people in different roles do not talk to one another, making collaboration difficult to realize.

SNA research suggests that denser networks (those that include individuals who are in more frequent, trusting, and diverse relationships) meet fewer challenges in establishing the norms or systemic practices needed to implement complex initiatives like those envisioned by WSCC.^30^ Educational reform research indicates that beyond network density, relationships that are horizontal (within a school) and vertical (across schools) are important for sustaining system‐wide change.^57^ In this study, both districts demonstrated some evidence of clustering by role, minimal cross‐school relationships, and network densities less than half of the ideal threshold.^31^ These characteristics indicate that WSCC implementation in these districts may be more challenging without interventions that open up information flows. One such intervention could be to leverage individuals identified as core influencers as conduits to individuals, stakeholder groups, or schools that are not yet connected. In SNA, individuals who bridge structural holes are “brokers.”^57^ Through a broker's “betweenness”—or position in‐between others—they have particular influence within their social network.

There are examples from the health and education sectors that demonstrate how SNA can be used to promote systems change.^33^, ^34^, ^35^, ^36^, ^37^, ^57^ Althabe et al.^36^ used SNA methods to survey providers and identify opinion leaders in a study on evidence‐based obstetrical practice implementation. Once identified, opinion leaders were trained on new best practices and how to communicate these new techniques to colleagues. Positive impacts of the study were observed and sustained 12 months after the intervention. It is also important to remember that just as individuals who hold brokering positions may facilitate the opening of social networks, so too can they hoard information, create boundaries, or exacerbate inequity.^57^ As with all interventions, the possibility of unintended consequences must be acknowledged so that systems and structures are built to resist these all‐too‐familiar imbalances.

Just as SNA provided study districts a more nuanced understanding of the people and relationships through which WSCC implementation might be possible, message testing helped to identify how school health leaders and champions might communicate with stakeholders to gain support to build a healthy school from the inside out. The use of health communication to generate behavior change is a hallmark of public health campaigns. Its use for the promotion of healthy schools and the concepts reflected by the WSCC model, specifically, is a relatively new area of research. Studies funded by RWJF as part of the larger THSS initiative found that stakeholders defined healthy schools as multidimensional, that there was broad support for healthy schools, and that messages promoting equity, student success, and the “whole child” resonated across audiences.^58^, ^59^ Many of these same message themes tested well in this study. Messages that emphasized empathy, teamwork, and action were also well‐received, especially when shared by trusted individuals through communication channels that aligned with stakeholder needs.

A key lesson across these studies is the importance of tailoring communication efforts to different audiences. One message might activate a teacher to join a wellness council while another encourages a parent to speak up at a board meeting on behalf of a school health initiative. In health communication, this tailoring is part of a process referred to as audience segmentation. Segmentation can occur along any meaningful demographic or personal characteristic. In our study, stakeholder role was used to differentiate messaging preferences. Exploration of segmentation across additional characteristics, such as length of tenure as an educator, may be beneficial for engaging with audiences and understanding their motivations, especially when the goal is to promote a new idea or behavior.

It is important to note that the lessons learned from this study stop short of measuring changes in or progress toward implementation of the WSCC model. Our aim was to explore new approaches to unpacking WSCC and to use our experience to develop a set of translational products that help bridge the gap between research and practice. Translational products, which share research in ways that non‐academic audiences can understand, are especially important in increasing the likelihood that research reaches practitioners and guides their real‐world application.^60^ The process of translation continues: Following the release of the toolkit, the team convened a learning community consisting of 11 school districts across the nation. The learning community allows for a more in‐depth assessment of how schools and districts of different sizes, geographies, and with different student populations use the toolkit to address their school health priorities.

## Limitations

In addition to the limitations mentioned above, data collection occurred over one academic year and with a limited number of schools and stakeholders. The team worked closely with liaisons to ensure that a diverse group was recruited, but it was not possible to include input from all stakeholders. Additionally, due to IRB and district policies, students participated only in group model building workshops.

As with any research, low response rates can bias results. This is particularly concerning for SNA, which relies on complete information for analytic procedures. Specifically, response rates below 80% may contribute to less accurate depictions of how information flows within a network.^61^ In this study, the response rates for districts were approaching adequate (between 57% and 69%), and findings are expected to be accurate. There may be several reasons for sub‐optimal response rates. For SNA, in particular, some respondents may have conflated the roster and network surveys and not understood that they were separate points of data collection. For others, the network survey took approximately 20–25 minutes to complete and requested what might be perceived as personal information (ie, frequency of contact with others, level of trust for others). Despite these limitations, novel and applicable information on conditions necessary for WSCC implementation were identified.

### IMPLICATIONS FOR SCHOOL HEALTH

Methods like SNA, message testing, and group model building require significant knowledge and human resources. Educators and school health practitioners may wonder how they would be able to use even one of these methods—let alone all 3—considering everything that is already on their plates. There is good news. First, these methods are complementary to steps recommended for WSCC implementation^19^, ^41^ and can be used at any point in the implementation process. Second, the Healthy Schools Toolkit breaks each method down into smaller, more manageable steps, and practitioners can choose from a range of activities that align to their needs and capacity.

- *Identify and leverage leaders and champions*. Many schools and districts use school health coordinators, advisory councils, and wellness teams to champion WSCC‐related efforts. Influential leaders and strong social networks are critical to ensuring the success of these efforts. Below are ways to integrate SNA into existing efforts to build a strong team and network. Additional details and templates to help you complete these steps are provided in Module 1 (People) of the Healthy Schools Toolkit.
  ◦ *If you only have 1 week*. Work with 10–12 colleagues to develop a roster of individuals in your school or district network. Those who receive more nominations are likely to have more influence.
  ◦ *If you have <1 month*. Survey individuals on the roster to understand how frequently they communicate with and are trusted by others.
  ◦ *If you have >1 month*. Create a map to visualize the flow of information through the social network and to identify facilitators and barriers to spreading information.
- *Communicate effectively*. There are many strategies to gain the support of stakeholders for WSCC, including being intentional about the messages shared and how they are shared. Below are options detailed in Module 3 (Messages) of the Healthy Schools Toolkit, which includes a variety of templates and supplementary resources.
  ◦ *If you only have 1 week*. Adopt the messengers, messages, and messaging strategies that have been found to be effective in other schools and districts. No additional research or tailoring needed!
  ◦ *If you have <1 month*. Conduct a few message testing interviews to identify which messengers and messaging strategies work for different audiences in your school or district.
  ◦ *If you have >1 month*. Conduct additional message testing interviews with 5–7 people to develop specific messages that resonate with different audiences in your school or district.
- *Change and align systems*. Coordinating policies, processes, and practices is no small feat. Group model building is one approach that has promising applications for WSCC implementation. To learn more about these applications, read Ballard et al's article and Module 2 (Systems) of the Healthy Schools Toolkit.

### Human Subjects Approval Statement

School districts approved all study protocols. The IRB of Washington University in St. Louis approved all research activities (IRB ID #201710098 and #201710099).

### Conflict of Interest

The authors declare no conflicts of interest.
